# Endocardial thickening in dilated cardiomyopathy associated with *FHL2* variant: a case report

**DOI:** 10.3389/fgene.2026.1841224

**Published:** 2026-10-02

**Authors:** Yana Wang, Lingfang Liang, Lingke Liu, Jiajia Ren, Xiaoxian Wang

**Affiliations:** 1 Department of Pediatrics, The Affiliated Hospital of Shaoxing University, Shaoxing, China; 2 Department of Pediatric Intensive Care Unit, Children’s Hospital, National Clinical Research Center for Child Health, Zhejiang University School of Medicine, Hangzhou, China

**Keywords:** case report, dilated cardiomyopathy, endocardial fibroelastosis, FHL2, heart failure

## Abstract

**Background:**

*FHL2* is critical for cardiac development, and its variants are associated with early-onset severe dilated cardiomyopathy (DCM), though such reports remain rare.

**Case presentation:**

The patient was a 30-day-old male infant admitted with a 3-day history of cough, a 2-day history of poor feeding, and lethargy lasting half a day. Echocardiography revealed left ventricular enlargement, with a minimum left ventricular ejection fraction (LVEF) of only 13%, and endocardial thickening. Genetic testing identified a heterozygous missense variant c.391C>T (p.Arg131Cys) in *FHL2*. This variant was confirmed to be *de novo*. According to the American College of Medical Genetics and Genomics (ACMG) guidelines, it was classified as likely pathogenic, suggestive of DCM. After heart failure management and other symptomatic treatments, the patient’s clinical symptoms improved. However, LVEF remained low at 15.5% during the 6-month follow-up.

**Conclusion:**

The heterozygous missense variant c.391C>T (p.Arg131Cys) in *FHL2* is associated with DCM in this patient, and the endocardial thickening may represent a secondary change. However, we acknowledge that without pathological examination of the explanted heart or endomyocardial biopsy, the possibility of primary endocardial fibroelastosis (EFE) cannot be completely excluded. This study emphasizes the clinical importance of distinguishing true primary EFE from secondary EFE-like endocardial changes associated with severe DCM, and genetic testing is valuable for their differential diagnosis.

## Introduction

Dilated cardiomyopathy (DCM) is a myocardial disease characterized by dilation of one or both ventricles and significant impairment of systolic function. It is the most common type of pediatric cardiomyopathy, accounting for approximately 50% of cases. Among infants under 1 year of age, the prevalence is as high as 8.34 per 100,000, and nearly 40% of affected children undergo heart transplantation or die within 2 years of diagnosis (Heymans et al., 2023; Malinow et al., 2024). DCM has complex and diverse etiologies, and pathogenic variants can be detected in some cases across multiple gene families, including those encoding sarcomeric proteins, cytoskeletal proteins, and ion channels (McNally and Mestroni, 2017). In recent years, with advances in molecular genetics research, the genotype-phenotype correlations of some pathogenic genes have been progressively clarified, providing an important basis for the early diagnosis and precision treatment of DCM in children (Njoroge et al., 2022).

*FHL2* is essential for cardiac development. The protein it encodes is involved in regulating the development of the complex cardiac structure and plays a significant role in the proliferation, differentiation, and migration of cardiomyocytes (Jiang et al., 2025). Previous studies have suggested that the loss or dysfunction of *FHL2* is closely associated with pathological myocardial remodeling and the development of heart failure (Tran et al., 2016; Li et al., 2024; Stathopoulou et al., 2022). Currently, only a few case reports of infantile DCM caused by *FHL2* variants have been published worldwide (Yu et al., 2023; Yamamoto et al., 2025). The association between these variants and clinical phenotypes remains to be further elucidated. This article reports a rare case of severe DCM with endocardial thickening in a male infant. We also systematically analyze the relationship between *FHL2* genotypes and phenotypes in conjunction with the literature, aiming to provide insights for the early diagnosis and precision treatment of pediatric DCM.

### Case presentation

History of Present Illness: The 30-day-old male infant was admitted to a local hospital with a 3-day history of cough, 2-day history of poor feeding, and half a day of lethargy, poor responsiveness, pallor, frothy sputum, and dyspnea. Initial vital signs were as follows: body temperature 35.0 °C, heart rate 150 beats/min, respiratory rate 50 breaths/min, blood pressure 83/36 mmHg, and peripheral oxygen saturation 70% on room air. Based on these findings, clinicians at the local hospital diagnosed severe pneumonia with heart failure and initiated the following management. After endotracheal intubation was performed, the infant received ceftriaxone, furosemide, milrinone and intravenous fluid resuscitation, then was emergently transferred to our hospital.

Birth and Family History: He was born at 37 + 4 weeks of gestation by cesarean section to a primiparous mother due to abnormal fetal heart rate. His birth weight was 3.45 kg (SD score: 0.74), appropriate for gestational age, and he had no edema. Routine prenatal fetal ultrasound was performed and showed no structural cardiac abnormalities. There was no history of asphyxia or resuscitation at birth. There was no family history of cardiomyopathy. The patient had no significant past medical history.

Physical Examination: Weight 4.1 kg, length 52 cm, head circumference 37 cm. On admission, the patient was comatose and pale, with a weak cry. Under mechanical ventilation (BiPAP mode, FiO_2_ 50%, PIP 25 mbar, PEEP 7 mbar, rate 35 breaths/min, Ti 0.55 s), vital signs were as follows: temperature 35.5 °C, heart rate 148 bpm, respiratory rate 60 bpm, and blood pressure 85/74 mmHg (mean arterial pressure 78 mmHg, measured non-invasively by cuff), and oxygen saturation 100%. Physical examination revealed sluggish pupillary light reflex under continuous sedation with midazolam, bilateral coarse breath sounds with wheezing and rales, and slightly muffled but regular heart sounds without pathological murmurs. Liver and spleen were not palpable and the neurological examination was negative. The extremities were cool, with a capillary refill time of approximately 3 s.

Laboratory Findings: White blood cell (WBC) count and C-reactive protein (CRP) were within normal limits. The following laboratory parameters were elevated: procalcitonin was 7.37 ng/mL, ferritin was >30,000 μg/L and B-type natriuretic peptide (BNP) was >5,000 pg/mL. Liver, renal, and coagulation functions were impaired. Blood culture, respiratory pathogen testing, and antinuclear antibodies were negative, and the inherited metabolic profile was unremarkable. On admission, arterial blood gas showed: pH 7.09, PaCO_2_ 75.4 mmHg, PaO_2_ 39.7 mmHg, base deficit −8.5 mmol/L, lactate 4.3 mmol/L, HCO_3_^−^ 21.8 mmol/L, indicating a mixed respiratory and metabolic acidosis with severe hypoxemia (Table 1).

**TABLE 1:** Laboratory findings at the time of admission.

Laboratory indicators	Test value	Reference range
Arterial pH	7.09	7.35–7.45
PaCO2 (mmHg)	75.4	35.0–48.0
PaO2 (mmHg)	39.7	>60
Base excess (mmol/L)	−8.5	−3.0–3.0
lactate (mmol/L)	4.3	0.5–1.6
HCO_3_^−^(mmol/L)	21.8	21.0–28.0
WBC (×10^9^/L)	8.02	4.00–12.00
Hemoglobin (g/L)	101	110–155
Platelet (×10^9^/L)	191	100–400
PCT (ng/mL)	7.37	0.07–0.35
CRP (mg/L)	1.20	0.00–6.00
Ferritin (μg/L)	>30,000	24.0–336.0
Troponin I (ng/mL)	0.456	<0.100
Myoglobin (ng/mL)	720.08	<100.02
CK-MB (ng/mL)	206.36	<5.85
BNP (pg/mL)	>5,000	<100
Total protein (g/L)	48.2	49.0–71.0
Albumin (g/L)	36.2	25.0–50.0
Total Bile acid (μmol/L)	2.5	0–10.0
Total Bilirubin (μmol/L)	96.1	≤17.0
Direct Bilirubin (μmol/L)	9.7	0.0–5.1
Indirect Bilirubin (μmol/L)	86.4	1.0–20.0
ALT (U/L)	108	<50
AST (U/L)	274	14–44
Creatinine (μmol/L)	50	13–33
LDH (U/L)	1516	110–295
APTT (seconds)	48.9	23–38
PT (seconds)	21.1	9–14
D-dimer (mg/L)	30.75	<0.55
Fibrinogen (g/L)	0.72	1.69–3.6
Blood culture	Negative	Negative
Respiratory pathogen nucleic acid	Negative	Negative
Sputum culture	Negative	Negative
Antinuclear antibody panel (20 items)	Negative	Negative
Metabolic profile	No specific abnormalities	No specific abnormalities
TORCH antibody	Negative	Negative

WBC: white blood cell; PCT: procalcitonin; CRP: C-Reactive Protein; CK-MB: creatine kinase isoenzyme; BNP: B-type Natriuretic Peptide; ALT: alanine aminotransferase; AST: aspartate aminotransferase; LDH: lactate dehydrogenase; APTT: activated partial thromboplastin time; PT: prothrombin time; Antinuclear antibody panel (20 items): antinuclear antibody, anti-double-stranded DNA, anti-nucleosome, anti-histone, anti-cardiolipin, anti-ribosomal P protein, anti-mitochondrial antibody M2, anti-neutrophil cytoplasmic antibody, anti-myeloperoxidase, anti-proteinase 3, anti-Sjögren’s syndrome-related antigen A (SSA)/Ro52, anti-SSA/Ro60, anti-Sjögren’s syndrome-related antigen B (SSB)/La, anti-topoisomerase I, anti-centromere protein B, anti-histidyl-tRNA, synthetase (anti-Jo-1), anti-U1-ribonucleoprotein, anti-nuclear autoantigen Sp-100, anti-promyelocytic leukemia protein, anti-smooth muscle antibody; TORCH: toxoplasma, rubella, cytomegalovirus, and herpes simplex virus types 1 and 2.

Imaging and Cardiac Assessment: Echocardiography (Figures 1A,B, and supplemental figures) revealed significant left ventricular enlargement: left ventricular end-diastolic diameter was 31 mm, with a body surface area-adjusted Z-score of +3.0. The aortic root diameter was 9 mm and the peak aortic valve flow velocity was 0.9 m/s, with no evidence of accelerated flow. There was severe reduction in left ventricular systolic function, with a left ventricular ejection fraction (LVEF) of 13% measured by the Simpson’s method. Severe left ventricular systolic dysfunction, left ventricular diastolic dysfunction, and mild-to-moderate mitral regurgitation together led to left atrial enlargement and an elevated mitral annular E/e′ratio (11.5), indicating elevated left atrial pressure. The increased left atrial pressure further resulted in elevated pulmonary venous pressure, which in turn led to pulmonary hypertension, supported by a tricuspid regurgitation peak pressure gradient of 41 mmHg and the presence of diastolic pulmonary regurgitation below the pulmonary valve. Echocardiography also revealed left ventricular posterior wall endocardial thickening of approximately 2 mm and a patent foramen ovale (2.6 mm in diameter). Four-hour dynamic electrocardiogram revealed sinus rhythm, occasional premature atrial contractions (5 beats), and ST-segment depression with T-wave inversion in leads II, III, aVF, and V4-V6 (Figure 1C). These non-specific electrocardiographic changes were likely related to increased ventricular workload and myocardial strain secondary to severe cardiac dysfunction, though impaired myocardial perfusion could not be definitively excluded. Chest X-ray showed cardiomegaly and left ventricular enlargement (Figure 1D), and cardiac computed tomography angiography revealed no abnormalities in the origin, course, or caliber of the coronary arteries. Ultrasound of the liver, gallbladder, spleen, and kidneys showed no abnormalities.

**FIGURE 1:**
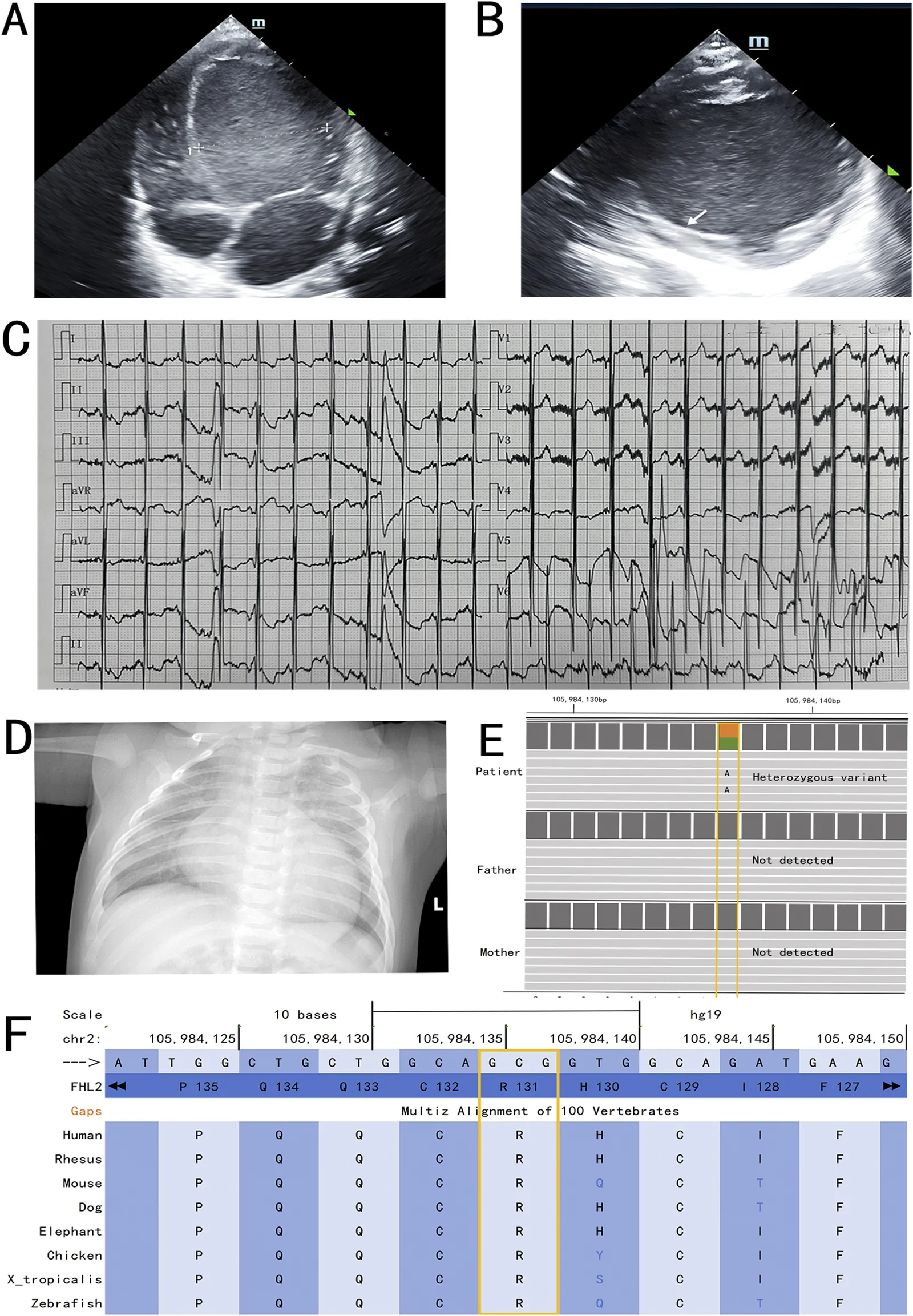
**(A)** Apical four-chamber echocardiogram demonstrating marked left ventricular dilation. **(B)** Parasternal short-axis echocardiogram at the papillary muscle level demonstrating 2 mm thickening of the left ventricular endocardium. **(C)** Electrocardiogram (ECG) shows ST-segment depression with T-wave inversion in leads II, III, aVF, V4 to V6. **(D)** Chest X-ray shows an increased cardiothoracic ratio. **(E)** Integrative Genomics Viewer (IGV) visualization of next-generation sequencing (NGS) results is shown. **(F)** The Arg131 residue of *FHL2* is highly conserved among vertebrates.

Genetic Analysis and Diagnosis: Blood samples were collected from the patient and his parents for trio-based whole exome sequencing. Genetic analysis revealed a *de novo* heterozygous missense variant in NM_001318895.3(*FHL2*):c.391C>T (p.Arg131Cys) (Variation ID: 48323, Accession: VCV000048323.11; OMIM: 602633). This variant was absent in both parents, confirming its *de novo* origin (Figure 1E). Based on the echocardiographic findings and genetic results, the patient was diagnosed with severe DCM associated with *FHL2* variant, with the endocardial thickening possibly interpreted as a secondary change.

Differential Diagnosis: The initial differential diagnoses included

1. Severe sepsis with septic cardiomyopathy: Although WBC and CRP were within normal limits, and blood culture and respiratory pathogen tests were negative, the patient presented with shock and multiorgan dysfunction, making this a key diagnostic consideration given the acute decompensation.
2. Primary endocardial fibroelastosis (EFE): The echocardiographic finding of left ventricular endocardial thickening raised the possibility of EFE, a common cause of infantile heart failure with similar imaging features.
3. Genetic DCM: The early age of onset, severity of systolic dysfunction, and lack of an identifiable acquired cause strongly suggested an underlying genetic etiology.
4. Viral myocarditis: Nucleic acid screening for respiratory pathogens including adenovirus, respiratory syncytial virus, influenza virus, and parainfluenza virus was negative. The patient had no typical prodromal symptoms of viral infection, and laboratory tests showed no evidence of acute viral infection; therefore, viral myocarditis was less likely.
5. Congenital infection-associated cardiomyopathy: Intrauterine infections such as congenital toxoplasmosis, rubella, cytomegalovirus, and herpes simplex virus can cause neonatal myocardial injury, ventricular dilation, and heart failure. We performed antibody testing, and all results were negative. No further testing such as PCR or viral culture was performed.
6. Mitochondrial disorders: Mitochondrial dysfunction can lead to dilated or hypertrophic cardiomyopathy in infancy, often accompanied by multisystem manifestations including elevated lactate, liver dysfunction, and neurological involvement. In this patient, the metabolic profile showed no specific abnormalities, which made a mitochondrial disorder unlikely.
7. Maternal autoantibody-mediated neonatal cardiomyopathy: Maternal autoantibodies such as anti-SSA/Ro and anti-SSB/La can cross the placenta and cause fetal/neonatal cardiomyopathy. In this case, the 20-item antinuclear antibody panel was negative, and the mother had no history of autoimmune disease; therefore, this possibility could be excluded.

### Treatment and clinical course:

#### Acute Phase resuscitation and vital support (Weeks 1–2)

A tiered respiratory support strategy was implemented, with the patient progressing from invasive to noninvasive mechanical ventilation over a period of 2 weeks. A combination regimen of inotropic agents (milrinone, digoxin), an angiotensin-converting enzyme inhibitor (captopril), and an aldosterone receptor antagonist (spironolactone) was administered for heart failure management, along with strict fluid restriction and diuresis using hydrochlorothiazide and furosemide. Multiorgan supportive care measures included cardioprotective therapy, hepatoprotection, anticoagulation, gastrointestinal protection, and anti-infective therapy. It should be noted that the presence of hyperferritinemia, markedly elevated cardiac enzymes, and severe heart failure at admission could not completely exclude the possibility of concurrent acute myocarditis. Therefore, methylprednisolone and intravenous immunoglobulin (IVIG) were administered empirically as immunomodulatory therapy, rather than as targeted treatment for the *FHL2* variant. Although some studies have suggested that such therapy may be beneficial in some children with DCM (Gagliardi et al., 2004), its efficacy in genetic DCM remains unclear. During the first week of intensive management, serum BNP markedly declined from >5,000 pg/mL to 510 pg/mL. By the second week, respiratory failure was completely resolved; hepatic, renal and coagulation laboratory parameters all recovered to normal limits. After sedatives were discontinued, the pupillary light reflex returned to normal.

#### Post-acute Phase maintenance therapy (Weeks 3–4)

Following hemodynamic stabilization, medications were gradually transitioned to oral formulations based on real-time assessment of cardiac function, heart rate, and blood pressure. However, echocardiography showed persistent significant left ventricular enlargement. Although left ventricular systolic function had improved compared with baseline (LVEF peaked at 22.5%), it remained severely reduced. The infant was discharged for outpatient rehabilitation with stable vital signs and complete resolution of clinical symptoms, alongside a customized long-term oral medication regimen.

#### Follow-up

The patient received IVIG injections every 2 months. The clinical condition was stable, with no dyspnea, cyanosis, or other discomforts. Feeding tolerance was acceptable, but the infant failed to thrive. At the 6-month follow-up, weight was 5.6 kg and length was 66.5 cm. Laboratory tests showed a BNP level of 432 pg/mL. Echocardiography revealed an LVEF of 15.5%, a left ventricular end-diastolic diameter of 40 mm (with a body surface area-adjusted Z-score of +3.2), and left ventricular posterior wall endocardial thickness of approximately 2.1 mm (Table 2).

**TABLE 2:** Clinical milestones of *FHL2*-related DCM patient.

Time point	Interventional measures	Major clinical outcomes
Week 1	Invasive mechanical ventilation, inotropic agents (milrinone, digoxin), captopril, spironolactone, furosemide, hydrochlorothiazide, fluid management, intravenous methylprednisolone and intravenous immunoglobulin, anti-infection therapy, and multi-organ function protection	The left ventricle remained significantly enlarged; BNP decreased from >5,000 to 510 pg/mL
Week 2	Non-invasive mechanical ventilation (oxygen via mask/nasal cannula); maintain acute phase treatment	Respiratory failure was corrected; liver and kidney function, as well as coagulation function, returned to normal
Weeks 3–4	Gradually adjust various medications to target doses of oral formulations: prednisone, digoxin, captopril, spironolactone, hydrochlorothiazide, coenzyme Q_10_, fructose diphosphate sodium, potassium chloride, vitamins A and D, and rivaroxaban	The patient remained clinically stable, and feeding was normal; The *FHL2* variant was confirmed to be *de novo* (c.391C>T); LVEF peaked at 22.5%
Discharge	Formulate an oral maintenance treatment plan; establish a regular follow-up plan	Vital signs were stable with no clinical symptoms; discharged for home rehabilitation
3-month follow-up	Routine follow-up (echocardiography, electrocardiogram, laboratory tests); adjust medication dosage; IVIG every 2 months	Weight was 5.4 kg and length was 64.0 cm. LVEF fluctuated between 16% and 19%, and BNP was approximately 450 pg/ml
6-month follow-up	Same as the 3-month follow-up regimen	Weight was 5.6 kg, length 66.5 cm, LVEF 15.5%, BNP 432 pg/ml, left ventricular diameter 40 mm, and endocardial thickness approximately 2.1 mm

BNP: B-type Natriuretic Peptide; LVEF: left ventricular ejection fraction.

## Discussion

The core clinical issue is an infant with early-onset severe DCM and endocardial thickening, associated with a *de novo* heterozygous missense variant in *FHL2* (c.391C>T, p.Arg131Cys), which necessitates a differential diagnosis from primary EFE. However, we acknowledge that the gene–disease evidence for *FHL2*-related DCM is still emerging, and no functional validation of this specific variant was performed in the present study. Through this case, we propose that endocardial thickening occurring in the setting of severe DCM is, in most instances, a secondary hemodynamic change rather than a primary endocardial disease. Genetic testing serves as an important component of the etiological assessment. Nevertheless, we acknowledge that although the identification of the pathogenic *FHL2* variant supports a genetic DCM as the underlying etiology in this case, the theoretical possibility of concomitant primary EFE cannot be completely excluded without histopathological confirmation from the explanted heart or an endomyocardial biopsy.

To begin with, we contrast the pathological and imaging features of secondary endocardial thickening against those of primary EFE. Primary EFE is pathologically characterized by diffuse proliferation of elastic and collagen fibers in the endocardium and is a common cause of heart failure in infancy. Its typical manifestations include marked left ventricular enlargement, global hypokinesis, decreased LVEF, as well as obvious endocardial thickening (often ≥2–3 mm) and prominent hyperechogenicity (Aldawsari et al., 2023; Xiao et al., 2022). In the present case, there was significant left ventricular dilatation, severely impaired systolic function, and endocardial thickening of 2 mm. Accumulating evidence indicates that in severe DCM, chronic elevation of left ventricular pressure and volume overload leads to increased wall stress, subendocardial hypoperfusion, and myocardial ischemia, which in turn induce secondary endocardial fibrotic remodeling (Barkhordarian et al., 2026). Such secondary changes can closely mimic primary EFE on echocardiography, making it difficult to distinguish them by imaging alone. Endocardial biopsy, although the diagnostic gold standard, is invasive and not routinely recommended.

Further distinctions can be drawn from echocardiographic characteristics, therapeutic response, and genetic evidence. The patient’s echocardiogram showed mild endocardial thickening of the left ventricular posterior wall, measuring 2 mm in thickness, along with severe left ventricular dilation and markedly reduced systolic function, but no left ventricular outflow tract obstruction was observed. During the 6-month follow-up, the endocardial thickening remained predominantly confined to the left ventricular posterior wall, and this localized distribution pattern tended to support that the lesion was secondary to hemodynamic changes. In contrast, EFE is characterized by dense, diffuse endocardial thickening (usually ≥2–3 mm) with markedly increased echogenicity, accompanied by varying degrees of left ventricular enlargement and systolic dysfunction (Xiao et al., 2022). EFE is associated with left ventricular outflow tract obstruction in some secondary cases (Gierlinger et al., 2025).

In terms of treatment response, our patient showed a slow increase in LVEF after aggressive heart failure management, consistent with the refractory course of DCM. Patients with primary EFE usually respond relatively well to diuretics and angiotensin-converting enzyme inhibitors (Sana and Mahajan, 2026).

Most importantly, genetic testing serves as a valuable complementary tool for etiological evaluation. This variant was classified as likely pathogenic based on three American College of Medical Genetics and Genomics (ACMG) criteria: confirmed *de novo* occurrence (PS2), absence or extremely low frequency in population databases (PM2_Supporting), and consistent predictions of deleterious effects by multiple *in silico* tools, including SIFT, PolyPhen-2, and MutationTaster (PP3) (Richards et al., 2015). The *FHL2* gene encodes the FHL2 protein (four-and-a-half LIM domains protein 2), which is a scaffolding protein predominantly expressed in the sarcomeres of cardiomyocytes, with almost no detectable expression in the endocardium, outflow tract, coronary vessels, or other cardiac regions (Kong et al., 2001). FHL2 interacts with other proteins primarily through its distinct LIM domains and participates in various cellular processes, including the regulation of gene expression, apoptosis, cell survival, cell migration, and signal transduction. In studies of DCM, FHL2 has been shown to directly bind to the N2B and Mis2 regions of titin, while simultaneously recruiting three metabolic enzymes—adenylate kinase, phosphofructokinase, and creatine kinase—to these sites, thereby providing energy for muscle contraction (Tran et al., 2016; Yu et al., 2023). However, the precise pathway through which the c.391C>T (p.Arg131Cys) variant is associated with DCM requires further investigation. Published data indicate that variants within LIM domains 0–2 are predominantly associated with DCM, whereas variants in the LIM3 domain correlate with hypertrophic cardiomyopathy (Yu et al., 2023). This observation suggests a possible link between the affected LIM domain and the clinical cardiomyopathy phenotype. However, this correlation is based on a very small number of reported cases and should be considered preliminary, requiring validation in larger independent cohorts (Supplementary Table 1). The variant in our case is located in the LIM2 domain of FHL2 protein, a region highly conserved across species (Figure 1F). To date, only two previously reported cases of infantile severe DCM carrying the same variant have been described worldwide (Table 3) (Yu et al., 2023; Yamamoto et al., 2025), both presenting with very early-onset heart failure and poor response to conventional therapy. Our case is the first to be associated with endocardial thickening. Notably, primary EFE can progress to DCM during the neonatal period, and several genetic etiologies for primary EFE have been identified (Luca et al., 2020). However, to the best of our knowledge, no cases of primary EFE caused by *FHL2* variants have been reported to date. The value of genetic testing lies in suggesting a genetic etiology for DCM and helping reduce the risk of misclassifying secondary endocardial thickening related to DCM as primary EFE.

**TABLE 3:** Summary of the present case and published cases of dilated cardiomyopathy associated with *FHL2* p.Arg131Cys variant.

Author	Year	Age at onset	Sex	Diagnosis	Treatment	Outcome
Yu C et al.	2023	1 month	Female	DCM	Oral medications: digoxin, captopril, metoprolol, furosemide, spironolactone, prednisone, vitamins B1, B2, C, levocarnitine, and fructose diphosphate sodium; no mechanical ventilation was documented	At 1 year and 7 months follow-up, cardiac ultrasound showed significant left ventricular enlargement with LVEF improving from 23.5% to 41.5%
Yamamoto H et al.	2025	Newborn	Male	Congenital DCM, cholestatic liver injury, and hyperferritinemia	Intravenous infusion: Catecholamines, milrinone, and furosemide; invasive mechanical ventilation	LVEF reached a nadir of 10%, increased to 40% at 1 month of age, but then deteriorated again, and the patient died at 2 months of age
Present case	2026	30 days	Male	DCM, endocardial thickening	Intravenous infusion: Milrinone, methylprednisolone, IVIG, combined with cardioprotective therapy, liver protection, anticoagulation, anti-infection therapy and fluid management; oral medications: digoxin, captopril, spironolactone, furosemide, hydrochlorothiazide; invasive mechanical ventilation	Clinical symptoms improved, but left ventricle remained enlarged; LVEF initially increased from 13% to 22.5% but then declined to 15.5% at the 6-month follow-up

DCM: dilated cardiomyopathy; LVEF: left ventricular ejection fraction; IVIG: intravenous immunoglobulin.

These differentiating criteria carry substantial clinical implications for patient management. Correctly distinguishing secondary endocardial thickening in DCM from primary EFE has direct clinical consequences: the treatment strategies differ. Some children with primary EFE may benefit from endocardial decortication (Barkhordarian et al., 2026), whereas the core treatment for *FHL2*-related DCM consists of heart failure medications, mechanical circulatory support, and evaluation for heart transplantation (Malinow et al., 2024). Our patient’s LVEF remained persistently below 20%. The patient carries a guarded long-term prognosis and requires early consideration for heart transplantation.

Therefore, we recommend that for infants with unexplained heart failure accompanied by endocardial thickening, clinicians should regard endocardial thickening as an important imaging clue to secondary remodeling due to DCM. Genetic testing (preferably trio whole-exome sequencing) should be promptly performed to assist in etiological workup, rather than limiting the diagnostic approach to primary EFE.

Lastly, several limitations of the present study should be acknowledged. This study is a single case report, and we did not perform *in vitro* or *in vivo* functional experiments to confirm the damaging effect of the p.Arg131Cys variant on FHL2 protein function. Most importantly, the lack of histopathological examination of the explanted heart or endomyocardial biopsy prevents us from definitively excluding primary EFE or confirming the nature of the endocardial thickening. Moreover, long-term follow-up data are limited, and more cases with extended follow-up are needed to clarify the epidemiology, prognosis and optimal treatment strategies.

## Conclusion

The clinical and genetic findings of this case have clear implications for the diagnosis and management of early-onset, severe cardiomyopathy. Based on this clinical case, it is clinically important to distinguish true EFE from secondary EFE-like endocardial changes associated with severe DCM, and genetic testing is valuable for their differential diagnosis. Nevertheless, histopathological examination remains the gold standard for definitive distinction.

## Data Availability

The raw data supporting the conclusions of this article will be made available by the authors, without undue reservation.
