# Supplementary material for: Endocardial thickening in dilated cardiomyopathy associated with FHL2 variant: a case report
Source: Front Genet. 2026 Oct 2;17:1841224. doi: 10.3389/fgene.2026.1841224 (PMC13673423; doi:10.3389/fgene.2026.1841224)
Supplement: Supplementary file 2 [file Supplementaryfile1.zip › supplementary material/Figure legends.docx]

**Figure legends**

**Figure 1** Apical four-chamber echocardiography showed significant left heart enlargement.

**Figure 2** Apical four-chamber echocardiography revealed pulmonary hypertension with a pressure gradient of 41 mmHg.

**Figure 3** Subcostal view echocardiography demonstrated a patent foramen ovale measuring 0.26 cm in diameter.

**Figure 4** Simpson's method from the apical four-chamber view showed severe left ventricular systolic dysfunction, with a markedly reduced left ventricular ejection fraction of only 13.04%.
